# A meta-analysis of the association between teacher-child interaction quality and young children’s social skills

**DOI:** 10.3389/fpsyg.2026.1808031

**Published:** 2026-07-09

**Authors:** Li Feng, Shuanglong Li

**Affiliations:** 1 College of Education Science, Kashi University, Kashi, China; 2 Member of Regional Basic Education Curriculum and Teaching Innovation Research Team, Kashi University, Kashi, China

**Keywords:** early childhood education, meta-analysis, social competence, teacher-child interaction quality, young social skills

## Abstract

Early childhood is a critical period for the development of children’s social skills. As a core element of early childhood education, teacher-child interaction quality is highly relevant, and variations in this quality are associated with the development of young children’s social skills. To synthesize the correlational evidence relevant to fostering the development of young children’s social skills, this study focused on the strength of the association between teacher-child interaction quality and young children’s social skills, as well as the moderating factors. Through systematic literature search and screening, 42 studies (42 effect sizes) were finally included, and a random-effects model was used for the meta-analysis. The results showed that there was a significant moderate positive correlation between teacher-child interaction quality and young children’s social skills (*r* = 0.21, 95% CI = [0.16, 0.26]). Tests of moderating effects revealed that the correlation between the two variables was significantly moderated by the measurement tool of teacher-child interaction quality and regional culture, but not by the sex ratio of young children, the mean age of young children, or the measurement tool of social skills. The observed positive correlation suggests that higher quality teacher-child interactions co-occur with stronger social skills in young children.

## 1 Introduction

Preschool children are innately curious and socially inclined, laying a foundation for the development of social skills such as empathy, communication, and cooperation (Pires et al., 2020). Kindergartens serve as the first formal setting where young children transition away from family protection and begin to develop into social beings. Kindergarten classrooms are therefore critical for fostering children’s social and learning skills (Hatfield et al., 2013; Hamre et al., 2014), with interactions among children, teachers, and peers being central to the acquisition of these skills (Williford et al., 2013b). The development of children’s social skills occurs through interactions with adults and peers within a socio-cultural context (Vygotskiĭ and Cole, 1978). Both positive and negative teacher-child interactions have been proven to influence young children’s social skills development and future academic achievement (Roorda et al., 2011), which highlights the critical role of teacher-child interaction quality in early education. Young children need to gradually acquire strategies for effective interaction with teachers and peers. On one hand, cooperation and communication skills enable children to express their ideas clearly and accurately during group learning, making them more likely to stand out, which in turn boosts self-efficacy (Hamre and Pianta, 2005). This self-efficacy becomes a sustained internal driver for learning, enabling children to adapt to the increasing demands of school readiness, and predicting their success in school and later in the workplace (Son and Chang, 2018). On the other hand, engaging in positive interactions through social skills such as empathy and friendly negotiation may reduce children’s aggressive behaviors (Perren and Alsaker, 2009). From a policy perspective in China, Education Modernization 2035 mandates that schools prioritize the cultivation of students’ communication, collaboration, and innovation skills to enhance their overall quality. Notably, intellectual development is defined not merely as the acquisition of knowledge, but also as the development of social skills such as communication and cooperation (Central Committee of the Communist Party of China, and The State Council of the People’s Republic of China, 2019).

With the growing global emphasis on early childhood education, numerous studies have identified teacher-child interaction quality in preschool settings as a proximal factor that plays an important role in the development of young children’s social skills (Pianta and Hamre, 2009). This quality exerts a significant impact on the development of social skills including communication, cooperation, responsibility, empathy, engagement, and self-control. A review of the existing literature reveals considerable inconsistencies in the findings regarding the association between teacher-child interaction quality and young children’s social skills. While most studies report a positive correlation (Stillerova, 2018), others suggest a negative association (Michelle Taylor, 2013), and some indicate no significant association (Karam, 2020). To date, no systematic synthesis or meta-analysis of the research in this field has been conducted. To resolve these academic debates and overcome the limitations of individual studies, which may be biased by factors such as participants’ nationality, children’s sex, and measurement tools, this study employs a meta-analytic approach to integrate previous findings. By examining the correlation and potential moderating variables, this research aims to provide more evidence for how teacher-child interactions may facilitate the development of social skills, thereby uncovering more generalizable and conclusive patterns.

### 1.1 The association between teacher-child interaction quality and young children’s social skills

Currently, three perspectives exist in the research on the association between quality of teacher-child interactions and young children’s social skills.

The first perspective posits a significant positive correlation between teacher-child interaction quality and young children’s social skills (Brophy-Herb et al., 2007). Attachment theory suggests that high-quality teacher-child interactions provide young children with emotional security for social interaction. Positive emotional support helps form a close emotional bond between teachers and children, and this bond may help children to develop prosocial relationships with others (Vygotskiĭ and Cole, 1978; Ainsworth, 2015). Relevant studies have also confirmed this view, with results indicating that positive teacher-child relationships strongly predict young children’s prosocial behavior and activity engagement (*r* = 0.55, *p* < 0.001) (Zhu J. et al., 2023). Vygotsky’s sociocultural theory further suggests that, based on the zone of proximal development, teachers provide scaffolded interactive guidance for the development of young children’s social skills, which may help children to acquire and internalize social skills within their zone of proximal development (Vygotskiĭ and Cole, 1978; Soininen et al., 2023). Researchers in China have found that the development of self-control in early childhood is largely associated with value internalization, which is in turn dependent on the influence of adults (Zhao, 2020). The ecological systems theory supports this view from a macro-environmental perspective. It posits that children develop through interactions with their surroundings, and once children enter kindergarten, teachers become significant others for young children (Howes and Hamilton, 1993). Teacher-child interaction is regarded as a core element of the microsystem (Pianta and Stuhlman, 2004). High-quality teacher-child interactions may help young children perceive teachers’ care and gain a sense of security (Spilt and Koomen, 2022), thereby facilitating the establishment of trusting relationships with teachers, which may serve as an important indicator of their environmental adaptation and social skills acquisition (Hamre and Pianta, 2001).

The second perspective suggests a negative correlation between teacher-child interaction quality and young children’s social skills. Correia et al. (2024) found a negative correlation between teacher-child interaction quality and young children’s social skills (*r* = −0.134). A third perspective posits that the correlation between teacher-child interaction quality and young children’s social skills is weak or non-significant. For instance, Broekhuizen et al. (2015) found that the correlation coefficients between teachers’ emotional support and young children’s social skills were −0.04 and 0.09 for children of different age groups. In another study, Stephens et al. (2023) reported no significant association between teachers’ emotional support and young children’s social skills, with a correlation coefficient of −0.01.

In summary, previous studies have reported inconsistent findings regarding the association between teacher-child interaction quality and young children’s social skills, and this association requires further investigation. Therefore, this study employs a meta-analytic approach to examine this association. Drawing on theoretical and empirical evidence, we propose the following hypothesis: there is a positive correlation between teacher-child interaction quality and young children’s social skills.

### 1.2 Moderating variables between teacher-child interaction quality and young children’s social skills

After reviewing existing literature, inconsistencies have been identified in the correlation between teacher-child interaction quality and young children’s social skills, suggesting that their association may be moderated by variables such as the sex ratio of children and the use of different measurement tools.

The sex ratio of young children may moderate the association between teacher-child interaction quality and young children’s social skills. This moderating effect may stem from sex differences in young children’s social skills. Boys and girls may differ in their developmental strengths in social skills, so variations in the sex ratio within a class may influence the association between teacher-child interaction quality and young children’s social skills (Mohamed, 2018). On the one hand, researchers have found that boys score lower than girls on the total score of all dimensions of social skills in early childhood (Salavera et al., 2020; Józsa et al., 2025). From the perspective of sex stereotypes, girls are expected to be more sensitive to others’ facial expressions (Briton and Hall, 1995). This belief may lead parents to prioritize fostering nonverbal sensitivity in girls (McClure, 2000). This may further contribute to girls outperforming boys in aspects such as self-awareness and perseverance (Yu et al., 2022), which may enable girls to identify teachers’ emotional signals more accurately during teacher-child interactions and, thus potentially form closer relationships with teachers more easily than boys (Brock and Curby, 2014). On the other hand, some scholars argue that sex has no significant effect on young children’s social skills, as teachers tend to believe that most social behaviors should be treated equally among boys and girls when assessing social skills (Smith et al., 2019). Studies have also found no significant differences in social skills between boys and girls in early childhood (*t* = 0.068, *p* > 0.05) (Li, 2020). Based on the above, Hypothesis 1 is proposed: the sex ratio of young children may moderate the correlation between teacher-child interaction quality and young children’s social skills.

Measurement tools for teacher-child interaction quality may moderate the correlation between teacher-child interaction quality and young children’s social skills. Currently, multiple measurement tools for teacher-child interactions have been developed in academia (Tobin, 2005), which have evolved from an emphasis on structural quality to process quality (Pianta and Hofkens, 2023). In the early stage of environmental structural quality assessment, Harms et al. revised the Early Childhood Environment Rating Scale (ECERS). However, studies have suggested that even with a high environmental score, children’s developmental outcomes may not be optimal if teacher-child interactions are not positive. Environmental indicators may only indirectly reflect teacher-child interaction quality, which may be associated with a weak correlation between measurement tools and children’s social skills. This has encouraged researchers to shift their focus to process quality (Cryer et al., 1999). The middle stage focused on relational quality assessment, with the Student-Teacher Relationship Scale (STRS) and the Closeness dimension of the Teacher-Child Relationship Scale (TCR Closeness) centering on the emotional bond and interaction quality of teacher-child relationships. These tools adopt a teacher self-report approach, measure three types of teacher-child relationships, and focus on the emotional connection between teachers and children. Self-report measures may be subject to common method bias, which could contribute to inconsistencies in the observed correlations (Hamre and Pianta, 2001). These two measurement tools have driven research to shift its focus from the learning environment to teacher-child relationships (Dede Yildirim et al., 2024). At present, the field has entered the stage of process quality assessment. The Classroom Assessment Scoring System (CLASS), developed by Mashburn et al. (2008), focuses on the assessment of three domains and ten dimensions of teacher-child interaction quality (Hamre et al., 2013). Based on the above, different types of measurement tools for teacher-child interactions differ significantly in data sources, assessed dimensions, and other aspects. Such differences may influence the association between these two variables. Accordingly, Hypothesis 2 is proposed: measurement tools for teacher-child interactions may moderate the correlation between teacher-child interaction quality and young children’s social skills.

Measures of social skills may moderate the associations between teacher-child interaction quality and young children’s social skills. The assessment methods for social skills have been continuously evolving, advancing from sole teacher-rated perspectives to multi-informant evaluations and in-situ behavioral observations (Green and Forehand, 1980). In the early stage, the Teacher–Child Rating Scale (TCRS), developed by Hightower et al. (1986), assesses children’s social skills from teachers’ perspectives, targeting children from kindergarten to primary school. It consists of four domains (task orientation, behavior control, self-confidence, and peer social skills) and 32 items (Hightower et al., 1986), but this measure may be susceptible to the subjective biases of teachers. To address the limitations of a single perspective, Gresham et al. developed the Social Skills Rating System (SSRS), which adopts a multi-informant evaluation approach and may reflect the development of young children’s social skills (Eslami et al., 2014). It is applicable to assessing social skills among students in preschool, primary, and secondary education, and the scale comprises three domains (cooperation, persistence, and self-control) with 40 items, enabling a holistic assessment of children’s social skills (Demaray et al., 1995). Subsequently, social skill measures have progressed toward more objective approaches. The Individualized Classroom Assessment Scoring System (inCLASS) emphasizes *in-situ* observations of children by trained professionals, who assess and code real-time interactions between teachers and children, as well as among peers in classroom settings (Booren et al., 2012). Composed of 10 dimensions, this measure effectively minimizes the subjectivity associated with informant reports (Downer et al., 2010). Based on the above, Hypothesis 3 is proposed: measures of social skills may moderate the correlation between teacher-child interaction quality and young children’s social skills.

Sample mean age may moderate the correlation between teacher-child interaction quality and young children’s social skills. Studies have shown that with an increase in sample mean age, the psychological and physical maturity of young children gradually improves, and the sociality of the child group also advances (Denham et al., 1991). Overall, groups with a higher mean age demonstrate higher levels of social skills among young children (Siekkinen et al., 2013). Stillerova (2018) conducted a longitudinal study and found that increasing sample mean age is a significant predictor of the development of young children’s social skills (the mean score of socio-emotional skills increased from 3.49 at T1 to 4.05 at T2), and older children demonstrate better performance in emotional regulation and peer cooperation skills. As sample mean age varies across studies, young children’s foundational levels of social skill development differ, which may contribute to discrepancies in the strength of the association between teacher-child interaction quality and social skills. Based on the above, Hypothesis 4 is proposed: sample mean age may act as a moderating variable in the correlation between teacher-child interaction quality and young children’s social skills.

Regional culture may moderate the correlation between teacher-child interaction quality and young children’s social skills. From a culture-specific perspective, the correlation between the two is not universal globally and varies significantly across countries and cultural backgrounds. For instance, teachers in European and American regions tend to emphasize empathy and active listening when interacting with young children, whereas teachers in East Asian regions often interact with children in a more implicit and reserved manner (Xu et al., 2023). Different interaction styles may promote the development of different dimensions of young children’s social skills. Meta-analyses on related topics have further indicated that regional cultural background may play a significant moderating role in the association between teacher-child interaction quality and young children’s social skills (Xu et al., 2024).

## 2 Research methods

### 2.1 Literature search and screening

This study selected two major Chinese core databases, CNKI and WanFang, as well as three authoritative international English databases: Web of Science, SpringerLink, and SAGE. As these databases are mainstream platforms in the fields of education and psychology, they may comprehensively cover the Chinese and English empirical literature required for this study. To avoid retrieval redundancy and literature overlap, no additional databases were included. To ensure a comprehensive and accurate search, this study centered its search on teacher-child interaction (including instructional support, classroom organization, and emotional support) and social skills (including communication, cooperation, self-control, etc.), combined with synonymous expressions and preschool-related keywords. Search terms were developed according to the theoretical connotation of core variables, and consistent search terms were used in both Chinese and English. All retrievals were restricted to title, abstract and keyword fields.

In English databases (including Web of Science, SpringerLink, and SAGE), the search strategy was formulated as follows: (“teacher-child interaction” OR “teacher children interaction”) AND (“social skills” OR “social competence” OR “communication” OR “cooperation” OR “assertion” OR “responsibility” OR “empathy” OR “engagement” OR “participation” OR “self-control”) AND (“preschool education” OR “early childhood education” OR “kindergarten”).

In Chinese databases (including the journal and master’s and doctoral dissertation databases of CNKI, WanFang journal and dissertation databases), the following Boolean search string was used: (“teacher-child interaction” OR “teacher-preschooler interaction” OR “teacher emotional support” OR “teacher classroom organization” OR “teacher instructional support”) AND (“social skills” OR “social competence” OR “communication” OR “cooperation” OR “assertion” OR “responsibility” OR “empathy” OR “engagement” OR “self-control”) AND (“preschool education” OR “early childhood education” OR “kindergarten”).

The literature screening process was conducted in two rounds following standard meta-analysis procedures. In the first round, the search results from both Chinese and English databases were initially screened based on titles and abstracts. Studies consistent with the research topic were retained for full-text evaluation. In the second round, full texts were reviewed according to the predetermined inclusion and exclusion criteria. Inconsistencies in coding were resolved by consulting the original literature and conducting multiple checks, after which the data analysis was finalized.

The present study focuses on the strength of the association between teacher-child interaction quality and children’s social skills. Pearson correlation was selected as the unified effect size index due to its wide applicability in correlational meta-analysis. To ensure the comparability of effect sizes across different studies, this method was applied consistently. To satisfy the independence assumption of meta-analysis, only one effect size per sample was retained.

We established clear inclusion and exclusion criteria in accordance with conventional meta-analysis guidelines: studies of children with special needs were excluded, and only typically developing children were included; duplicate publications based on the same dataset were also excluded, and only one entry was retained from the same dataset. The language of included literature was limited to Chinese and English.

**FIGURE 1:**
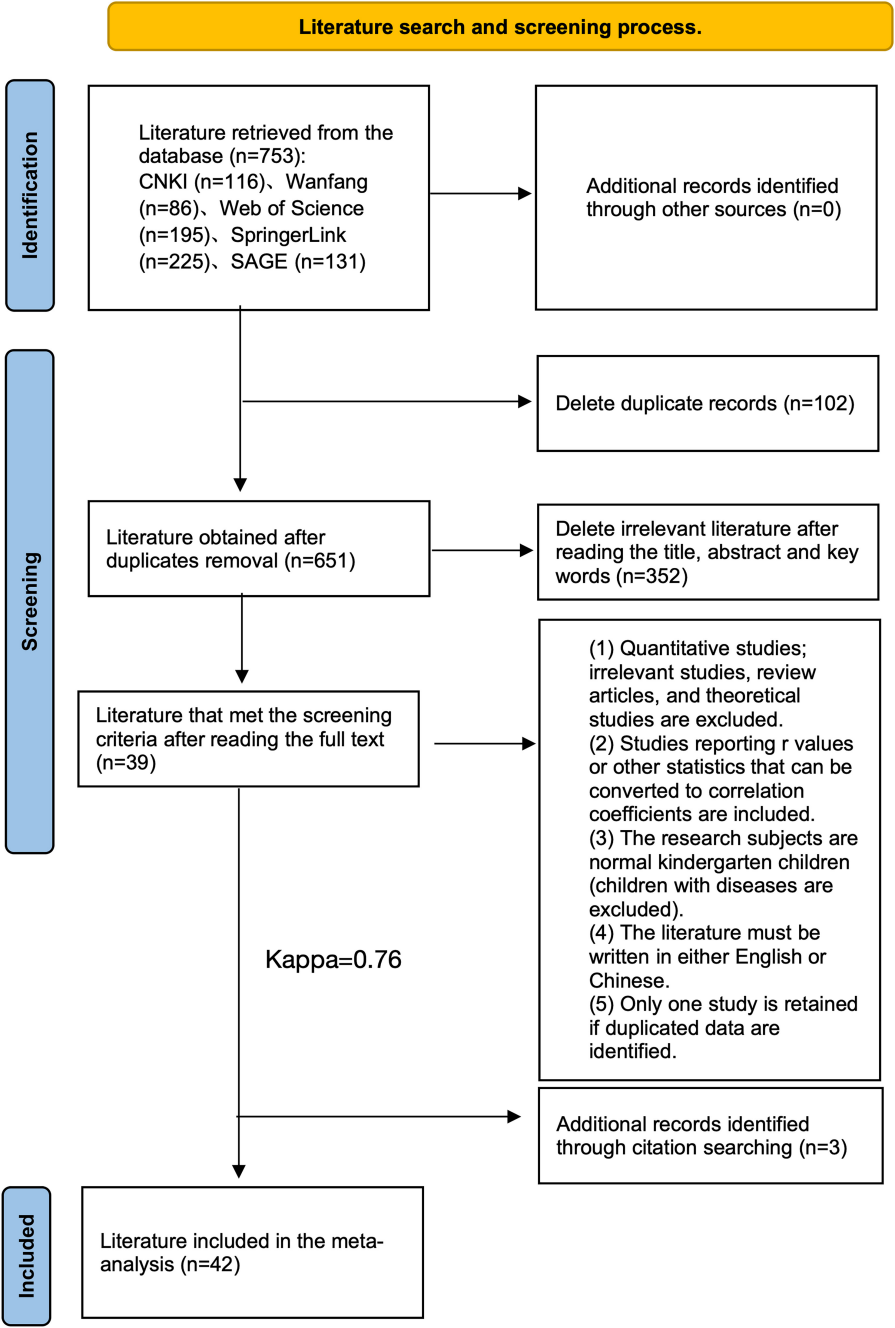
PRISMA flow diagram.

Following the PRISMA flow diagram (Figure 1), we retrieved a total of 753 records from five databases (CNKI, Wanfang, Web of Science, SpringerLink, and SAGE). After removing 102 duplicates, 651 records remained. Then, 352 irrelevant records were excluded after screening the titles, abstracts and keywords, leaving 299 records eligible for full-text assessment. Full-text screening was performed on the remaining records, and 39 studies met the initial inclusion criteria after full-text review. Subsequently, 3 additional records were identified through citation searching. Finally, a total of 42 studies were included in the meta-analysis (corresponding to 42 independent effect sizes, as only one effect size was retained per study to ensure statistical independence). Sample sizes for moderator analyses varied across subgroups due to missing data and the removal of subgroups with fewer than three studies to ensure statistical power, consistent with recommendations by Song et al. (2014).

Two independent reviewers conducted the literature screening and coding in parallel. Any discrepancies between the two reviewers regarding study eligibility, data extraction, or coding were resolved through discussion until consensus was reached. If a disagreement persisted, a third senior reviewer was consulted to make the final decision. Inter-coder reliability was assessed using Cohen’s kappa coefficient, with a value of 0.76, indicating excellent agreement between the two independent reviewers (Landis and Koch, 1977).

The publication time of the included literature ranged from January 1, 2006 to July 1, 2025. The detailed screening process is presented in Figure 1: Flowchart of Study Screening.

### 2.2 Literature coding and effect size conversion

During the literature coding and data processing stage, the unit of analysis was the independent effect size. The data structure of this study was a two-level nested structure of studies and effect sizes, with most studies including only one effect size and a few studies containing multiple effect sizes.

We applied three aggregation rules based on the number of dimensions within the same construct: if a study reported only one effect size for the core construct, this single effect size was used directly; if a study reported fewer dimensions under the same construct, the arithmetic mean was used for aggregation; and if a study reported more dimensions under the same construct, the dependent effect size aggregation formula proposed by Hunter–Schmidt was used for synthesis and correction (Hunter and Schmidt, 2004). In this way, multiple dependent effect sizes within the same study were integrated into one independent overall effect size to satisfy the independence assumption of meta-analysis.

This study adopted the following effect size extraction rules: the correlation coefficient between teacher-child interaction quality and young children’s social skills after controlling for other variables was extracted first; if this was not reported, the zero-order correlation coefficient was extracted; if neither was available, the β-value was extracted and converted into a unified Pearson correlation coefficient using the corresponding formula.

Each study was coded according to the following categories: title, first author and publication year, nationality of the sample, mean age, sample size, correlation coefficient, measurement domains and tools of teacher-child interactions, and measurement domains and tools of social skills (Table 1).

**TABLE 1:** Basic information of included literature.

References	*N*	Geographic region	Mean age	TCI tool	SS tool	r (k)	Aggregation method
Stillerova, 2018	848	Europe	6.17	CLASS	PBRS	0.170, 1	Arithmetic mean
Mashburn et al., 2008	2307	Europe	4.00	CLASS	TCRS	0.089, 1	Arithmetic mean
Wang et al., 2021	388	East Asia	4.07	CLASS	SSIS	0.320, 1	Single effect size
Baldanza, 2013	714	Europe	4.13	CLASS	inCLASS	0.091, 1	Arithmetic mean
Mohamed, 2018	160	Middle East	4.83	STRS	SSRS	0.440, 1	Hunter aggregation
Broekhuizen et al., 2015	545	Europe	2.25	CLASS	BITSEA	−0.040, 1	Arithmetic mean
Ana, 2016	180	Europe	5.31	CLASS	SSRS	0.070, 1	Hunter aggregation
Curby et al., 2013	1758	Europe	4.62	CLASS	TCRS	0.100, 1	Single effect size
Burchinal et al., 2008	759	Europe	4.52	ECERS CLASS	TCRS	0.060, 1	Arithmetic mean
Hoffman, 2016	2377	Europe	3.82	CLASS	SSRS	0.100, 1	Single effect size
Vitiello et al., 2022	1358	Europe	5.59	STRS	TCRS	0.280, 1	Arithmetic mean
Karam, 2020	218	Europe	–	M-ORCE	CBQ	0.510, 1	Single effect size
Dede Yildirim et al., 2024	185	Europe	4.00	TCR	SCBE	0.126, 1	Single effect size
Stephens et al., 2023	550	Europe	4.75	CLASS	SSRS	−0.010, 1	Single effect size
Burchinal et al., 2010	1129	Europe	–	CLASS	TCRS	0.138, 1	Single effect size
Diebold and Perren, 2022	86	Europe	3.64	CLASS	SOCOMP	0.042, 1	Arithmetic mean
Correia et al., 2024	336	Europe	5.01	CLASS	SSRS	−0.090, 1	Arithmetic mean
Burchinal et al., 2021	362	Europe	4.54	CLASS	TCRS	0.110, 1	Single effect size
Pakarinen et al., 2020	441	Europe	6.13	CLASS	MASCS	0.320, 1	Arithmetic mean
Awadalla, 2019	688	Europe	1.91	CLASS	ASQ	−0.050, 1	Single effect size
Whittaker et al., 2024	1826	Europe	5.59	CLASS	TCRS	0.020, 1	Single effect size
Siekkinen et al., 2013	1268	Europe	6.13	CLASS	MASCS	0.021, 1	Arithmetic mean
Brock and Curby, 2014	2645	Europe	–	STRS	TCRS	0.360, 1	Arithmetic mean
Brophy-Herb et al., 2007	183	Europe	4.23	CRS	TCRS	0.510, 1	Single effect size
Li, 2020	570	East Asia	5.13	CLASS	SSIS	0.246, 1	Arithmetic mean
Zhuang, 2019	334	East Asia	–	CLASS	CSS	0.023, 1	Single effect size
Yu et al., 2022	243	East Asia	–	CLASS	CSQ	0.518, 1	Hunter aggregation
Wu, 2023	446	East Asia	–	CLASS	CSS	0.459, 1	Hunter aggregation
Zhu J. et al., 2023	484	East Asia	5.56	STRS	CBS	0.550, 1	Single effect size
Rimm-Kaufman et al., 2009	172	Europe	5.41	CLASS	CSS	0.150, 1	Hunter aggregation
Williford et al., 2013a	605	Europe	4.18	CLASS	inCLASS	0.270, 1	Single effect size
Zhao, 2018	135	Europe	4.58	STRS	CEQ	0.533, 1	Arithmetic mean
Elizarov et al., 2024	300	Middle East	5.73	STRS	SDQ	0.320, 1	Single effect size
Wang et al., 2022	198	East Asia	4.90	STRS	SSRS	0.380, 1	Hunter aggregation
Budrevich, 2019	411	Europe	4.81	CLASS	SSIS	0.038, 1	Hunter aggregation
Tanya et al., 2021	126	–	4.73	TCRS	SSIS	0.380, 1	Arithmetic mean
Sette et al., 2013	88	Europe	4.90	STRS, 2001	SCBE	0.400, 1	Single effect size
Serena, 2025	1068	Europe	4.98	STRS	BASC	0.180, 1	Single effect size
Liu et al., 2024	378	East Asia	1.84	STRS	SCBE	0.260, 1	Single effect size
Tutkun and Eskidemir Meral, 2025	382	Middle East	2.56	STRS	SSIS	0.470, 1	Single effect size
Wu et al., 2022	532	East Asia	4.29	STRS	TRSSA	0.273, 1	Arithmetic mean
Slot and Bleses, 2018	184	Europe	4.33	inCLASS	inCLASS	−0.170, 1	Single effect size

N, sample size; TCI tool, teacher-child interactions measurement tool; SS tool, social skills measurement tool; CLASS, Classroom Assessment Scoring System; STRS, Student–Teacher Relationship Scale; ECERS, Early Childhood Environment Rating Scale; M-ORCE, Mother–Child Observation Rating Scale for Early Childhood; TCR, Teacher-Child Relationship Scale; CRS, classroom rating scale; CRS, child rating scale. A dash (–) indicates that the relevant data was not reported in the original study. Abbreviations for social skills measurement tools are as follows: PBRS, Preschool Behavior Rating Scale; TCRS, Teacher-Child Relationship Scale; SSIS, Social Skills Improvement System; inCLASS, Individualized Classroom Assessment Scoring System; SSRS, Social Skills Rating System; BITSEA, Brief Infant-Toddler Social and Emotional Assessment; CBQ, Children’s Behavior Questionnaire; SCBE, Social Competence Behavior Evaluation; SOCOMP, Social Competence Scale; MASCS, Multidimensional Assessment of Social Competence; ASQ, Ages and Stages Questionnaires; CSS, Child Social Skills Scale; CSQ, Classroom Social Competence Questionnaire; CBS, Child Behavior Scale; CEQ, Classroom Engagement Questionnaire; SDQ, Strengths and Difficulties Questionnaire; TRSSA, Teacher Rating Scale of Social Adjustment; BASC, Behavior Assessment System for Children. r, Pearson correlation coefficient; k, number of effect sizes; Hunter aggregation, Hunter and Schmidt (2004) reliability correction and aggregation.

For correlation coefficient coding, if a study did not report the correlation coefficient between teacher-child interaction quality and social skills but provided the β-value, these statistics were first converted into correlation coefficients (r) using the corresponding formulas $r={\{}_{\beta \times 0.98+0.05,\beta \geq 0}^{\beta \times 0.98-0.05,\beta <0}$ before coding:

Where:

*r* = converted correlation coefficient for meta-analytic synthesis

β = standardized regression coefficient reported in the primary study (Card, 2012).

In addition, if the original literature did not report the overall correlation coefficient between teacher-child interaction quality and young children’s social skills but presented the correlation coefficients between teacher-child interactions and each subdimension of social skills instead, the overall correlation coefficient between teacher-child interaction quality and young children’s social skills was synthesized using the following formula before coding:


$$r_{{\mathrm{xy}}_{b}}=\frac{\sum r_{i}\,r_{j}}{\sqrt{n+n\,\left(n-1\right)\,\bar{r_{x_{i}\,x_{j}\,\sqrt{m+m\,\left(m-1\right)\,\bar{r_{y_{i}\,y_{j}}}}}}}}$$

Where:

*r*_*xyb*_ = aggregated independent correlation coefficient between teacher-child interaction quality (*x*) and children’s social skills (*y*)

*r*_*x_i_y_y_*_ = zero-order correlation coefficient between the i-th dimension of teacher-child interaction and the corresponding i-th dimension of social skills

*n* = number of dimensions of teacher-child interaction included in the study

*m* = number of dimensions of social skills included in the study

$\bar{r_{x_{i}\,x_{j}}}$ = mean intercorrelation between different dimensions of teacher-child interaction

$\bar{r_{y_{i}\,y_{j}}}$ = mean intercorrelation between different dimensions of children’s social skills

(Hunter and Schmidt, 2004)

This step converted multiple dependent effect sizes from the same study into one independent effect size per study, which is consistently reported in Table 1 and used in all subsequent meta-analytic analyses.

Detailed information on original effect sizes, aggregation rules, calculation procedures, and how dependent effect sizes were converted into one independent effect size per study is provided in Supplementary Table 1. Supplementary Tables 2–8 contain the details of included studies, coding schemes, quality evaluation and reliability data.

The number of effect sizes (k) varies across moderator analyses for two key reasons: some included studies failed to report the relevant moderator variable, and subgroups with fewer than three studies were excluded to ensure sufficient statistical power (Song et al., 2014).

### 2.3 Control and test of publication bias

In this study, both published academic journal articles and unpublished master’s and doctoral dissertations (from the CNKI database) were included in the literature selection process, which is a common strategy to help mitigate publication bias to a certain extent. For the assessment of publication bias, four complementary statistical methods were selected for comprehensive evaluation: funnel plots, Egger’s regression test, Rosenthal’s fail-safe N, and Duval and Tweedie’s trim-and-fill method. Each method serves a distinct function to ensure robust and reliable bias detection. The reason for applying multiple methods simultaneously is that the funnel plot is a visual tool that judges publication bias by the degree of dispersion of effect sizes. A symmetric distribution of effect sizes indicates low publication bias with a minor impact on the results of the meta-analysis. However, the funnel plot is somewhat subjective. Rosenthal’s fail-safe N was further used to quantify the stability of the meta-analytic results against potential publication bias. Therefore, Egger’s regression test was additionally employed to analyze the linear regression results: a non-significant result suggests the absence of publication bias, while potential bias requires the trim-and-fill method, which involves estimating and supplementing the missing studies to adjust the overall effect size.

### 2.4 Model selection

At present, there are two models for effect size synthesis: the fixed-effects model and the random-effects model. The fixed-effects model is based on the assumption that all included studies measure a single underlying overall effect size, with between-study heterogeneity being theoretically non-existent or negligible. The discrepancy between the observed effect size and the true effect size in the studies is solely attributed to the random sampling error of research samples. By contrast, the random-effects model posits that each study has its own true effect size, which is not a fixed value but drawn from a broader population distribution. In other words, the difference between the observed value of a study and the overall mean stems from both within-study sampling error and true between-study heterogeneity.

Based on an extensive literature review, this study argues that factors such as young children’s sex ratio, mean age and measurement tools may influence the strength of the association between teacher-child interaction quality and young children’s social skills. Thus heterogeneity assessment was conducted prior to model selection. Meanwhile, the Q statistic and I^2^ index were examined. The I^2^ index represents the proportion of between-study variation in effect sizes relative to the total variation. Specifically, I^2^ ≤ 50% indicates low heterogeneity, ranging from 50% to 75% denotes moderate heterogeneity, and I^2^ > 75% signifies high heterogeneity, following widely accepted meta-analytic criteria. If the Q statistic is statistically significant and I^2^ > 75%, the random-effects model is adopted, as it fits heterogeneous data better; otherwise, the fixed-effects model is used for analysis.

### 2.5 Data processing

Pearson’s correlation coefficient r was adopted as the effect size index in this study, as it is suitable for reflecting linear association between two continuous variables. The software Comprehensive Meta-Analysis (CMA) version 3.3 was used to conduct the publication bias test, heterogeneity test, main effect analysis, and moderator variable analysis due to its reliable analytical functions for meta-analysis. Two approaches were applied to analyze moderator variables: subgroup analysis was used for categorical moderators, while meta-regression analysis was employed for continuous moderators, which matches the conventional analytical paradigm for different moderator types. In line with existing studies (Song et al., 2014), the number of effect sizes in each group was set to no fewer than three to ensure sufficient statistical validity.

## 3 Results

In the data coding process, Pearson’s correlation coefficient r and sample sizes were input into Comprehensive Meta-Analysis (CMA) version 3.3 software. The software was used to convert the effect sizes into *Fisher’s Z* scores and their corresponding variances, along with other relevant indicators. A meta-analysis of the overall effects of each variable was then conducted based on the generated analytical results.

### 3.1 Heterogeneity test

A heterogeneity test was performed on the effect sizes in this study to determine whether the fixed-effects model or the random-effects model should be adopted, and whether meta-regression analysis or subgroup analysis was required. The results of the heterogeneity test showed that *Q* = 780.02 (*p* < 0.001), df = 41, I^2^ = 94.74%, which exceeded the 75% criterion proposed by Huedo-Medina et al. (Higgins et al., 2003), indicating a substantially high level of heterogeneity. The 95% prediction interval was considerably wider than the confidence interval, reflecting high dispersion of effect sizes across studies. Given the high between-study heterogeneity, the random-effects model was more appropriate for the subsequent analyses in this study. The analysis results are presented in Table 2. Although significant heterogeneity existed, all included studies in this research focused on the core topic of the association between teacher-child interaction quality and young children’s social skills. Moreover, strict adherence to the pre-specified inclusion and exclusion criteria ensured conceptual and methodological comparability across studies. The pooled analysis can provide the overall effect direction for this field, and further sources of heterogeneity can be explored through subsequent subgroup analysis or meta-regression. Thus, performing a pooled analysis under the current high heterogeneity is still theoretically and empirically justified methodologically.

**TABLE 2:** Results of heterogeneity test.

Outcome variable	Heterogeneity				95% CI		Tau-squared	
	Q	*df*	*p*	*I* ^2^	Lower	Upper	Tau-squared	Standard error
Teacher-child interactions	780.02	41	0.000	94.74	0.16	0.26	0.03	0.01
Emotional support	211.25	21	0.000	90.06	0.07	0.16	0.01	0.11
Classroom organization	121.51	10	0.000	91.77	0.06	0.23	0.02	0.01
Instructional support	198.14	11	0.000	94.45	0.08	0.26	0.02	0.01

CI, confidence interval; df, degrees of freedom; Q, Cochran’s Q statistic; I^2^, heterogeneity index; Tau-squared, between-study variance. Heterogeneity was assessed using the *Q*-test and I^2^ statistic. A *p*-value < 0.05 indicates significant heterogeneity.

### 3.2 Publication bias test

**FIGURE 2:**
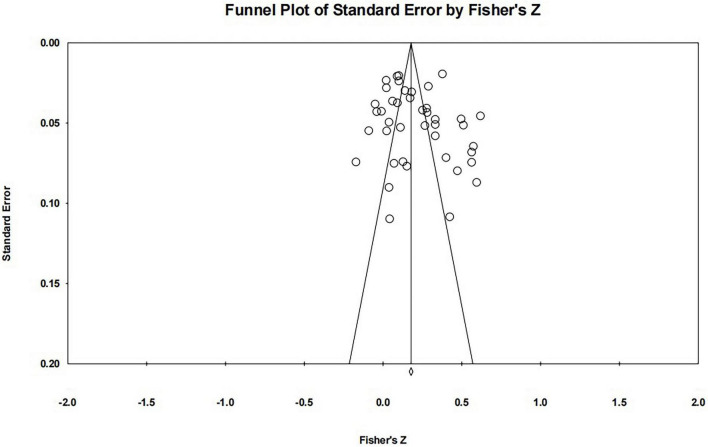
Funnel plot.

**FIGURE 3:**
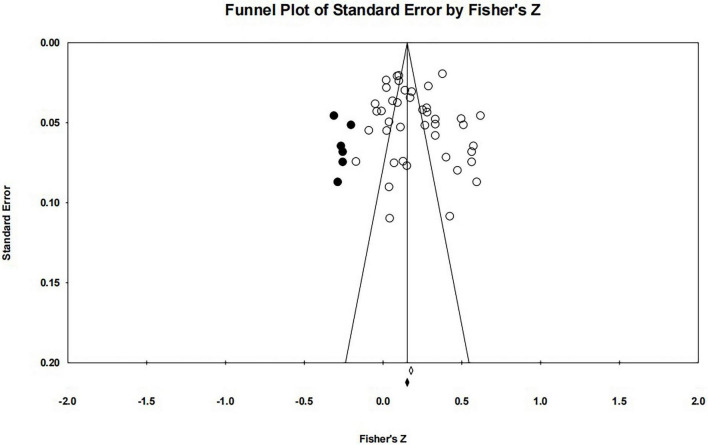
Funnel plot with trim-and-fill method.

To identify and quantify potential publication bias, a publication bias test was conducted on all effect sizes in this study. To ensure the accuracy and objectivity of test results, a variety of complementary statistical methods were adopted sequentially. First, a preliminary judgment was made via a funnel plot. The funnel plot (Figure 2) showed that the effect sizes were concentrated at the top of the inverted funnel plot and distributed relatively symmetrically on both sides of the overall effect, with no obvious visual asymmetry. Second, to account for the subjectivity of the funnel plot, Egger’s regression test was performed (see Table 3). The estimated intercept of the regression equation in Egger’s test was 2.28, with a 95% confidence interval of [−0.87, 5.42]. The two-tailed *p*-value for the intercept was 0.15, which was non-significant at conventional levels. Additionally, the fail-safe number analysis indicated that 9,482 missing studies with null results would be required to render the current significant pooled effect non-significant (*p* > 0.05), a value far higher than the critical threshold of 5k + 10 (220 when *k* = 42). This suggests that the pooled effect is relatively robust against potential missing studies. Finally, to further corroborate the robustness of the results, the trim-and-fill method was applied (Figure 3). The trim-and-fill analysis identified 6 missing studies on the left side of the funnel plot. However, the adjusted pooled correlation coefficient under the random-effects model remained statistically significant: *r* = 0.16, with a 95% confidence interval of [0.10, 0.21], which was consistent in direction and similar in magnitude to the original effect size (*r* = 0.21, 95% CI = [0.16, 0.26]).

**TABLE 3:** Results of Egger’s intercept test for publication bias.

Intercept	SE	95% CI lower limit	95% CI upper limit	*t*-value	*df*	*P*-value (one-tailed)	*P*-value (two-tailed)
2.28	1.56	−0.87	5.42	1.46	40	0.08	0.15

CI, confidence interval; SE, standard error; df, degrees of freedom. Egger’s intercept test was conducted to assess publication bias. A *p*-value < 0.05 indicates statistically significant publication bias.

### 3.3 Main effect test and sensitivity analysis

A total of 42 independent effect sizes were included in the present study under the random-effects model to estimate the correlation strength between teacher-child interaction quality and young children’s social skills (Table 4). The pooled correlation coefficient (*r*) was 0.21, with a 95% confidence interval of [0.16, 0.26]. Among the dimensions of teacher-child interactions, the correlation coefficient between teachers’ emotional support and young children’s social skills was 0.12 (*p* < 0.001), that between teachers’ classroom organization and young children’s social skills was 0.15 (*p* < 0.001), and that between teachers’ instructional support and young children’s social skills was 0.17 (*p* < 0.001). The correlations for each dimension and the overall analysis in this study were all statistically significant, as shown in Table 4. According to the criteria proposed by Cohen (Muller, 1989), an *r*-value of 0.1 indicates a low correlation, 0.2 a moderate correlation, and 0.3 a high correlation. Based on this standard, the results suggest a significant, moderate positive correlation between the two variables, indicating that improved teacher-child interactions may positively promote the development of young children’s social skills.

**TABLE 4:** Results of main effect test.

Outcome variable	*k*	*r*	Sensitivity analysis	95% CI	*z*-value	*Q*	*df*	*p*	*I* ^2^
Teacher-child interactions	42	0.21	0.20∼0.22	[0.16, 0.26]	7.92	780.02	41	0.000	94.74
Emotional support	22	0.12	0.10∼0.13	[0.07, 0.16]	4.72	211.25	21	0.000	90.06
Classroom organization	11	0.15	0.11∼0.17	[0.06, 0.23]	3.38	121.51	10	0.000	91.77
Instructional support	12	0.17	0.12∼0.19	[0.08, 0.26]	3.69	198.14	11	0.000	94.45

k, number of included studies; r, pooled correlation coefficient (effect size); 95% CI, 95% confidence interval; *z*-value, test statistic for the significance of the pooled effect size; Q, Cochran’s Q statistic for heterogeneity test; df, degrees of freedom; *p*, *p*-value for the significance of the pooled effect size and heterogeneity test; I^2^, index of heterogeneity (percentage of total variation due to between-study heterogeneity); Sensitivity analysis, range of pooled effect sizes after leave-one-out sensitivity analysis.

**FIGURE 4:**
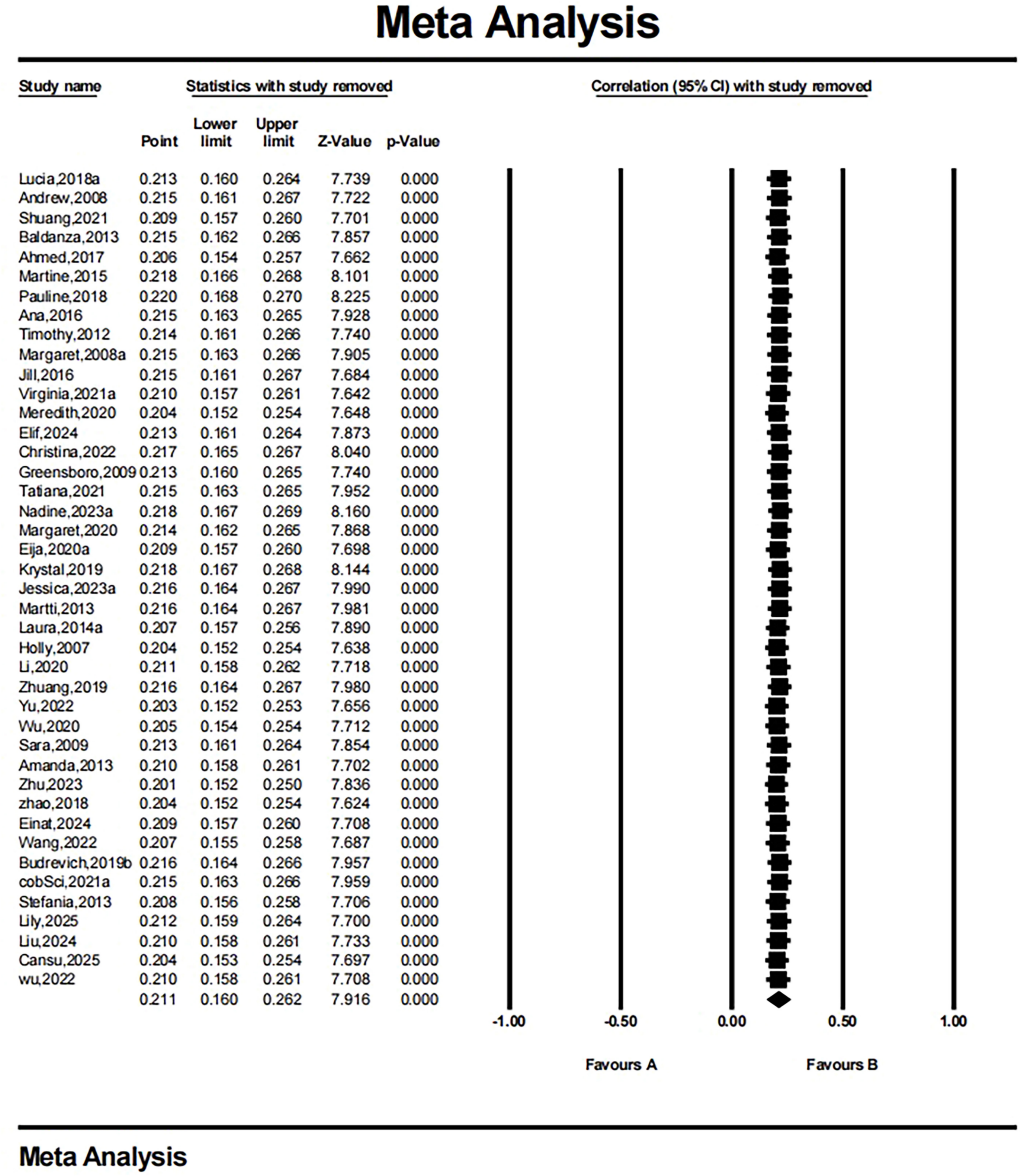
Sensitivity analysis plot.

To verify the stability and reliability of the pooled effect results, a sensitivity analysis was conducted by removing one study at a time to examine the robustness of the results. The pooled effect sizes after omitting each individual study fluctuated between 0.20 and 0.22, with all 95% confidence intervals excluding zero and all *p*-values less than 0.001 (Figure 4). All effect sizes and their corresponding confidence intervals were relatively concentrated; no significant outliers were observed, and the overall direction of the results did not change substantially due to any single study.

In this study, the Appraisal Tool for Cross-Sectional Studies (AXIS) (20 items in total) was used to assess the risk of bias among the 42 included studies (Downes et al., 2016). According to the AXIS criteria, studies with fewer than 4 high-risk items were classified as low risk of bias, those with 4–7 items as moderate risk, and those with 8 or more items as high risk. The results showed that 39 studies were rated as low risk of bias, 3 as moderate risk, and no studies were rated as high risk. To examine the robustness of the results, a sensitivity analysis was performed by excluding the 3 moderate-risk studies and recalculating the pooled effect size. The pooled correlation between teacher-child interaction quality and young children’s social skills was *r* = 0.18, 95% CI [0.14, 0.23], *p* < 0.001, which was consistent in direction with the original results (*r* = 0.21, 95% CI [0.16, 0.26]). This indicates that the conclusions of this study are robust and reliable.

### 3.4 Moderator effect test

Subgroup analysis and meta-regression analysis were employed according to the type of moderator variables, to examine the moderator variables of the association between teacher-child interaction quality and young children’s social skills, with the results as follows:

First, the moderating effect of the sex ratio of young children on the association between the quality of teacher–child interactions and young children’s social skills was not significant. As child sex ratio was a continuous variable, meta-regression was adopted accordingly. Only 41 effect sizes were included in the moderation analysis for child sex ratio, as relevant data were not reported in a subset of included studies. Meta-regression analysis (41 effect sizes) showed that the regression coefficient of the proportion of males on the effect size was not significant (*b* = −0.17, Standard Error = 0.74, *z*-value = −0.24, 95% CI = [−1.62, 1.27], *p* = 0.81), and the overall model test was also not significant (*Q* = 0.06, df = 1, *p* = 0.81). Hypothesis 1 was not supported.

Second, the measurement tool of teacher-child interaction quality significantly moderated the correlation between teacher-child interaction quality and young children’s social skills. As assessment tools were categorical variables, subgroup analysis was performed. The subgroup analysis included 35 effect sizes, as subgroups with fewer than 3 studies were excluded following the recommendations of Song et al. (2014) to guarantee statistical validity. Subgroup analysis (35 effect sizes) showed that under the mixed-effects model, the pooled correlation coefficient was *r* = 0.24, with a 95% CI = [0.19, 0.28], *Q* = 21.32, *p* < 0.001. Hypothesis 2 was supported. The subgroup analysis by reporting method included 41 effect sizes, as one study did not report the relevant assessment method. Further subgroup analysis by reporting method (41 effect sizes) showed a pooled effect size of *r* = 0.22, 95% CI = [0.18, 0.26], *p* < 0.001. The correlation for teacher-reported studies (*r* = 0.37, 95% CI = [0.30, 0.43]) was significantly higher than that for observer-rated studies (*r* = 0.15, 95% CI = [0.10, 0.20]), which may suggest that the measurement method is an important source of heterogeneity in effect sizes, as shown in Table 5; the derivation of the analytic sample is presented in Table 5.1.

**TABLE 5:** Moderation analysis of the relationship between teacher-child interaction quality and children’s social skills.

Moderator variable	Subgroup	*k*	*r*	95% CI		Two-tailed test	
				Lower limit	Upper limit	*z*	*p*
Social skills	inCLASS	3	0.07	−0.14	0.28	0.69	0.492
	CSS	3	0.22	−0.09	0.49	1.40	0.160
	SSIS, 2008	5	0.24	0.07	0.39	2.79	0.005
	SSRS, 1990	5	0.16	0.01	0.31	2.06	0.040
	TCRS, 1986	9	0.19	0.09	0.28	3.71	0.000
T-C interactions	SCBE	3	0.25	0.12	0.38	3.59	0.000
	CLASS, 2008	23	0.15	0.09	0.21	4.72	0.000
	STRS, 1992	4	0.44	0.33	0.54	7.09	0.000
	STRS, 2001	8	0.30	0.22	0.37	7.11	0.000
Reporting method	Observer report	29	0.15	0.10	0.20	5.20	0.000
	Teacher report	12	0.37	0.30	0.43	10.20	0.000
Regional culture	East Asia	9	0.35	0.24	0.45	5.91	0.000
	Europe and America	29	0.15	0.10	0.20	5.37	0.000
	Middle East	3	0.41	0.31	0.50	7.36	0.000

CI, confidence interval; SE, standard error; I^2^, heterogeneity index; k, number of included studies; r, pooled correlation coefficient; z, *z*-test statistic; T-C, Teacher-Child; inCLASS, Classroom Assessment Scoring System for Inclusive Settings; CSS, Child Social Skills Scale. SSIS, Social Skills Improvement System; SSRS, Social Skills Rating System; SCBE, Social Competence and Behavior Evaluation; TCRS, Teacher-Child Relationship Scale; CLASS, Classroom Assessment Scoring System; STRS, Student-Teacher Relationship Scale; SSRS, Social Skills Rating System; Variations in the number of effect sizes across subgroup and meta-regression analyses are due to missing moderator data and the exclusion of subgroups containing less than 3 studies (Song et al., 2014).

**TABLE 6:** Derivation of analytic samples across moderator analyses.

Moderator variable	Analysis type	Total effect sizes	Included effect sizes	Exact exclusion reason
Young children’s sex ratio	Meta-regression	42	41	1
Young children’s mean age	Meta-regression	42	36	2
Social skills measurement tool	Subgroup analysis	42	28	3
Teacher-child interaction tool	Subgroup analysis	42	35	3
Reporting method	Subgroup analysis	42	41	4
Regional culture	Subgroup analysis	42	41	5

1 = One effect size was excluded from meta-regression analysis due to missing child sex ratio data; 2 = Six effect sizes were excluded from meta-regression analysis due to missing mean age data; 3 = Subgroups containing fewer than three effect sizes were excluded from subgroup analysis; 4 = One effect size was excluded due to unspecified assessment method (unclear whether teacher-reported or observer-rated); 5 = One effect size was excluded due to unclear regional cultural information.

Third, the measurement tool of social skills did not moderate the association between teacher-child interaction quality and young children’s social skills. The subgroup analysis included 28 effect sizes, as subgroups with fewer than 3 studies were excluded following the recommendations of Song et al. (2014) to ensure reliable statistical outcomes. Subgroup analysis (28 effect sizes) showed a pooled correlation coefficient of *r* = 0.19, 95% CI = [0.13, 0.25], with a between-group Q statistic of 2.49 and *p* = 0.78. The result was not statistically significant, and Hypothesis 3 was not supported, as shown in Table 5; the derivation of the analytic sample is presented in Table 5.1.

Fourth, young children’s mean age did not exert a moderating effect on the association between teacher-child interaction quality and young children’s social skills. Since age was a continuous moderator, meta-regression analysis was applied. As relevant data were not reported in some studies, the meta-regression was based on 36 effect sizes. Meta-regression analysis (36 effect sizes) indicated that the regression coefficient of mean age on the effect size was not significant (*b* = 0.02, Standard Error = 0.03, *z*-value = 0.80, 95% CI = [−0.03, 0.07], *p* = 0.42). The overall model test was also not significant (*Q* = 0.65, df = 1, *p* = 0.42). Hypothesis 4 was not supported.

Fifth, regional culture significantly moderated the correlation between teacher-child interaction quality and young children’s social skills. Regional culture was categorical, so subgroup analysis was conducted. The subgroup analysis included 41 effect sizes, as one study did not report relevant regional cultural information. Subgroup analysis (41 effect sizes) under the mixed-effects model showed a pooled correlation coefficient of *r* = 0.22, 95% CI = [0.18, 0.27], with a Q statistic of 24.45 and *p* < 0.001. Further analysis showed differences in the correlation across cultural contexts: studies conducted in East Asia (*r* = 0.35, 95% CI = [0.24, 0.45]) and the Middle East (*r* = 0.41, 95% CI = [0.31, 0.50]) showed significantly stronger correlations than those in Europe and the United States (*r* = 0.15, 95% CI = [0.10, 0.20]). This may indicate that regional culture is an important source of effect-size heterogeneity, supporting Hypothesis 5, as shown in Table 5; the derivation of the analytic sample is presented in Table 5.1.

## 4 Discussion

### 4.1 The association between teacher-child interaction quality and young children’s social skills

Inconsistencies have existed in previous findings regarding the association between teacher-child interaction quality and young children’s social skills, and no comprehensive clarification has been provided by existing studies to date. This study adopted a meta-analytic approach to synthesize existing studies on the two variables, and found a significant moderate positive correlation between them (*r* = 0.21, *p* < 0.001). Specifically, there was a weak positive correlation between teacher emotional support and young children’s social skills (*r* = 0.12, *p* < 0.001), a weak positive correlation between teacher classroom organization and young children’s social skills (*r* = 0.15, *p* < 0.001), and a weak positive correlation between teacher instructional support and young children’s social skills (*r* = 0.17, *p* < 0.001). This finding reveals a significant positive association between teacher emotional support and young children’s social skills, which provides empirical support for attachment theory, as secure emotional bonds are theoretically associated with children’s social skill levels (Sroufe, 2005). Teacher instructional support is also consistent with sociocultural theory and ecological systems theory. Scaffolded interactions and environmental factors are associated with young children’s social skill profiles within their zone of proximal development, and environmental factors also play a role in this process. Overall, these results are consistent with the conclusions of many previous studies that have reported a positive correlation between teacher-child interaction quality and young children’s social skills. The observed positive association indicates the statistical relevance of teacher-child interaction quality in relation to young children’s social skills. The findings also clarify inconsistencies regarding the effect magnitude of the correlation between the two variables. This study did not support the viewpoint that there is a high correlation between teacher-child interactions and social skills (Brophy-Herb et al., 2007), nor did it support an extremely weak positive correlation (Whittaker et al., 2024), no significant correlation, or even a negative correlation between them (Correia et al., 2024). The correlation coefficient obtained in this study is relatively close to those reported in previous meta-analyses on similar topics (Li et al., 2024).

This study did not provide empirical support for the second viewpoint that there is a negative correlation between teacher-child interaction quality and young children’s social skills. Although previous studies have reported such a negative correlation (Broekhuizen et al., 2015), the present study did not support this view based on the current findings. Several reasons may account for the reported negative correlation in individual studies. First, the assessment of young children’s social skills in these studies was predominantly based on teacher reports, which reflect teachers’ subjective perceptions formed through repeated observations of children’s prosocial behaviors rather than objective direct measures of such behaviors (Correia et al., 2024). Second, the short time interval of measurements may have limited the detection of significant changes in children’s social skills (Jaggy et al., 2023), which may introduce potential errors in the measurement results. Additionally, while overly teacher-dominated curricula may suppress young children’s initiative in social interactions (Reeve, 2009), which theoretically could correspond to a negative correlation between the two variables, such extreme forms of teacher-child interactions accounted for an extremely small proportion in previous research. For these reasons, the claim of a negative correlation between teacher-child interaction quality and young children’s social skills lacks sufficient empirical support.

The findings of this study also did not provide empirical support for the third viewpoint, as the analysis confirmed a statistically significant correlation between teacher-child interaction quality and young children’s social skills. Although a small number of studies found no significant correlation between the two, this may be due to the fact that both measurement tools in those studies were based on teacher reports (Stephens et al., 2023).

### 4.2 Analysis of moderator effects

The conclusions drawn from this meta-analysis do not negate the findings obtained in some empirical studies. Rather, this meta-analysis only examined the bivariate correlation between teacher-child interaction quality and young children’s social skills, and other moderators may exist in their association. This study pre-registered child sex ratio, measurement tools for teacher-child interaction quality and social skills, and child mean age as moderators. To further explore effect heterogeneity, an exploratory analysis was also conducted to examine the moderating role of regional culture. The results showed that:

First, the association between teacher-child interaction quality and young children’s social skills was not moderated by the young children’s sex ratio, which did not support Hypothesis 1. Attachment theory suggests that young children’s need for a sense of security with teachers is stable across sex. This emotional need does not vary by the sex ratio within the class; that is, regardless of sex, young children all desire attention from teachers and hope to form close relationships with them (Partee et al., 2022). Only a small number of boys may exhibit aggressive behaviors due to individual characteristics. Such behaviors are not attributable to sex stereotypes but reflect individual differences (Kodak and Güzel, 2024). Therefore, it does not affect the cross-sex stability of young children’s need for security from teachers. Previous studies have indicated that the young children’s sex ratio moderates the correlation between the two variables (Mohamed, 2018). The present study found no moderating effect of young children’s sex ratio. This discrepancy may stem from the small sample sizes used in previous research, whereas this study examined the moderating role of young children’s sex ratio based on a considerably larger sample, which is more likely to reveal general patterns. The results showed that young children’s sex ratio did not significantly moderate the correlation between the two variables.

Secondly, this study found that measurement tools for teacher-child interaction significantly moderate the association between teacher-child interaction quality and young children’s social skills. Subgroup analyses examining the moderating effects of different measurement tools all yielded significant results (*p* < 0.001). When the CLASS instrument was used, the correlation coefficient between teacher-child interaction quality and young children’s social skills was 0.14 (*p* < 0.001), whereas it increased to 0.44 (*p* < 0.001) when the STRS developed in 1992 was employed. This difference may be attributed to the varying scopes of application of the tools. For instance, the CLASS instrument covers multiple educational stages, while the STRS was specifically designed for kindergarten settings. In comparison, the dimensions and behavioral definitions of the STRS may better align with the early childhood stage, whereas the universal design of CLASS may weaken its ability to precisely capture changes in young children’s behaviors (Al-Hendawi et al., 2025). On the other hand, the moderating effect of measurement tools is associated with rater identity. When using instruments such as the STRS, the quality of teacher–child interaction is rated based on teachers’ self-perceptions, yielding a correlation of *r* = 0.37 between teacher interaction quality and children’s social skills; this may introduce common method bias due to subjective judgment. By contrast, instruments such as the CLASS rely on observer reports, which tend to be more objective than self-reports, with a smaller correlation of *r* = 0.15. These findings further suggest that measurement approach may represent an important source of heterogeneity in the observed moderating effects.

In addition, Hypothesis 3 was not supported in this study: assessment tools for social skills did not significantly moderate the association between teacher-child interaction quality and young children’s social skills. This suggests that the association between these two variables is stable and robust, and does not vary substantially with different social skill measures, informants, or rating approaches. Although different measurement tools may differ in dimensional structure and items, they are generally consistent in focusing on young children’s social skills as the core construct (Zhu Z. et al., 2023). Differences between tools appear insufficient to alter the strength of the association. Thus, the association between teacher-child interaction quality and young children’s social skills is not significantly moderated by the tools used to assess social skills.

Furthermore, the average age of young children did not moderate the correlation between teacher-child interaction quality and young children’s social skills (*p* > 0.05), which is inconsistent with the results of previous meta-analyses (Li et al., 2024). From the perspective of attachment theory, children’s need for social interaction remains stable across early developmental stages (Howes et al., 1988). Teachers represent an important source of secure attachment for children in preschool settings (Beckh and Becker-Stoll, 2016; Ansari et al., 2025). The emotional support and instructional support provided by teachers are consistent and stable for children aged 3–6 years (Verschueren and Koomen, 2012; Curby et al., 2013). Such consistency may be associated with no significant differences in the association between teacher-child interaction quality and young children’s social skills across age groups, thereby weakening the moderating role of age in the association.

Finally, an exploratory analysis revealed that regional culture significantly moderated the association between teacher-child interaction quality and young children’s social skills, providing support for Hypothesis 5. The correlation was significantly stronger in East Asian and Middle Eastern samples than in European and American samples, which may reflect differences in educational values across cultural backgrounds. This finding is consistent with the notion of cultural specificity. Relevant studies indicate that in traditional collectivist cultures such as those in the Middle East, social values emphasize responsibility and harmony, and such contexts are linked to the development of prosocial behaviors including cooperation and empathy in young children (Alghamdi, 2023). In contrast, individualist cultures in Europe and the United States prioritize personal independence, and teachers’ active listening and communication support the development of social skills such as self-control and proactive social engagement (Chen et al., 2019).

### 4.3 Limitations

Previous studies have yielded inconsistent findings regarding the association between teacher-child interaction quality and young children’s social skills. The present meta-analysis identified a significant, moderate positive correlation between the two constructs; however, this study is not without limitations. First, this study only examined five moderating variables: measurement tools for teacher-child interaction quality, measurement tools for social skills, young children’s sex ratio, children’s age group, and cultural region. Other potential moderators, such as children’s family socioeconomic status, maternal educational level, teachers’ academic qualifications, and years of teaching experience, require further examination. Second, during literature screening, this study only included empirical studies that reported Pearson correlation coefficient r. This may result in the omission of representative theoretical papers, and thus influence the estimated correlation between the two variables. In addition, to ensure the reliability and stability of the results when analyzing the moderating effects of measurement tools, subgroups with fewer than three effect sizes were excluded in the subgroup analysis. Therefore, whether the association between teacher-child interaction quality and young children’s social skills is moderated by infrequently used assessment tools remains to be confirmed in future research. In addition, this study only included children with typical development and excluded those with special educational needs or developmental disorders. Therefore, the findings cannot be generalized to children with special needs such as autism or attention deficit hyperactivity disorder. Furthermore, the samples included in this study are mainly from Europe, America, the Middle East, and East Asia. Limited by geographical distribution and cultural backgrounds, the results should be applied with caution in regions with different cultural contexts or levels of economic development. Moreover, a variety of measurement tools were used to assess teacher-child interaction quality and children’s social skills, which differ in reliability, construct dimensions, and informant sources. When data on both teacher-child interaction quality and social skills were obtained from the same informant, common method bias may have inflated the observed correlation between the two variables. Finally, this study found very high heterogeneity, with I^2^ = 94.74% and a 95% prediction interval of [−0.12, 0.54], which is much wider than the 95% confidence interval. This suggests that effect sizes may vary considerably in future research on the association between teacher-child interaction quality and young children’s social skills, and negative effect sizes are even possible. Accordingly, the generalizability of the conclusions should be interpreted cautiously in light of specific research contexts.

## 5 Conclusion

This study systematically reviewed empirical research on the association between teacher-child interaction quality and young children’s social skills. A meta-analysis of 42 independent empirical studies revealed a stable and significant association between the two variables. The present study indicated that teacher-child interaction quality was significantly and moderately positively correlated with young children’s social skills. This association was significantly moderated by the measures of teacher-child interactions and regional culture, but not by children’s sex ratio, measurement tools of social skills, or mean age. These findings highlight the important value of high-quality teacher-child interactions in relation to young children’s social skills. Future studies investigating the association between teacher-child interaction quality and young children’s social skills should adopt appropriate and valid measurement tools.

## Data Availability

Publicly available datasets were analyzed in this study. This data can be found here: The datasets analyzed during the current study are available from the corresponding author on reasonable request.

## References

[B1] Ainsworth M. D. S. (2015). *Pa terns of Atachment: a Psychological Study of the Strange Situation.* Milton Park: Taylor & Francis Group. 10.4324/9780203758045

[B2] Alghamdi A. A. (2023). Culture in early childhood education: insights into saudi preschool teaching. *J. Educ. Learn.* 17 431–440. 10.11591/edulearn.v17i3.20804

[B3] Al-Hendawi M. Hussein E. Darwish S. (2025). Direct observation systems for child behavior assessment in early childhood education: a systematic literature review. *Discov. Ment. Health* 5 21–42. 10.1007/s44192-025-00139-z 39994157 PMC11850679

[B4] Ana L. V. (2016). *Teacher-Child Interactions and Children’s Social Skills and Problem Behaviors ECEC Dosage and Disability Status as Moderators.* Fairfax, VA: George Mason University.

[B5] Ansari A. Buckley M. N. Woods S. C. Gottfried M. (2025). The cumulative, timing-specific, and enduring associations between student–teacher relationships and early elementary outcomes. *Child Dev.* 96 475–491. 10.1111/cdev.14177 39563483 PMC11868690

[B6] Awadalla B. (2019). *Examining the Role of Varying Levels of Classroom Quality for Toddlers in Early Head Start and Subsidized Child Care Programs: Understanding Threshold Effects.* Coral Gables, FL: University of Miami, 1–91.

[B7] Baldanza M. T. (2013). *Teacher-Child Interactions and Children’s Peer Engagement in Pre-Kindergarten.* Oakland, CA: University of California, 1–81.

[B8] Beckh K. Becker-Stoll F. (2016). Formations of attachment relationships towards teachers lead to conclusions for public child care. *Int. J. Dev. Sci.* 10 103–110. 10.3233/DEV-16197

[B9] Booren L. M. Downer J. T. Vitiello V. E. (2012). Observations of children’s interactions with teachers, peers, and tasks across preschool classroom activity settings. *Early Educ. Dev.* 23 517–538. 10.1080/10409289.2010.548767 25717282 PMC4337404

[B10] Briton N. J. Hall J. A. (1995). Beliefs about female and Male nonverbal communication. *Sex Roles* 32 79–90. 10.1007/BF01544758

[B11] Brock L. L. Curby T. W. (2014). Emotional support consistency and teacher–child relationships forecast social competence and problem behaviors in prekindergarten and kindergarten. *Early Educ. Dev.* 25 661–680. 10.1080/10409289.2014.866020

[B12] Broekhuizen M. L. Aken M. A. G. V. Dubas J. S. Mulder H. Leseman P. P. M. (2015). Individual differences in effects of child care quality: the role of child affective self-regulation and gender. *Infant Behav. Dev.* 40 216–230. 10.1016/j.infbeh.2015.06.009 26210737

[B13] Brophy-Herb H. E. Lee R. E. Nievar M. A. Stollak G. (2007). Preschoolers’ social competence: relations to family characteristics, teacher behaviors and classroom climate. *J. Appl. Dev. Psychol.* 28 134–148. 10.1016/j.appdev.2006.12.004

[B14] Budrevich A. (2019). *Examining Classroom Quality as a Moderator between Pre-Kindergarten Participation and School Readiness.* West Lafayette, IN: Faculty of Purdue University, 1–18.

[B15] Burchinal M. Garber K. Foster T. Bratsch-Hines M. Franco X. Peisner-Feinberg E. (2021). Relating early care and education quality to preschool outcomes: the same or different models for different outcomes? *Early Child. Res. Q.* 55 35–51. 10.1016/j.ecresq.2020.10.005

[B16] Burchinal M. Howes C. Pianta R. Bryant D. Early D. Clifford R. et al. (2008). Predicting child outcomes at the end of kindergarten from the quality of pre-kindergarten teacher–child interactions and instruction. *Appl. Dev. Sci.* 12 140–153. 10.1080/10888690802199418

[B17] Burchinal M. Vandergrift N. Pianta R. Mashburn A. (2010). Threshold analysis of association between child care quality and child outcomes for low-income children in pre-kindergarten programs. *Early Child. Res. Q.* 25 166–176. 10.1016/j.ecresq.2009.10.004

[B18] Card N. (2012). *Applied Meta-Analysis for Social Science Research.* London: Routledge.

[B19] Central Committee of the Communist Party of China, and The State Council of the People’s Republic of China. (2019). *China’s education modernization 2035. Government of the People’s Republic of China.* Available online at: https://www.gov.cn/xinwen/2019/02/23/content_5367987.htm (accessed February 23, 2019).

[B20] Chen M. Zee M. Koomen H. M. Y. Roorda D. L. (2019). Understanding cross-cultural differences in affective teacher-student relationships: a comparison between dutch and Chinese primary school teachers and students. *J. Sch. Psychol.* 76 89–106. 10.1016/j.jsp.2019.07.011 31759472

[B21] Correia N. Carvalho H. Aguiar C. (2024). Does participation benefit children’s socio-emotional development? Positive associations between children’s participation and self-concept, through children’s perceptions. *Early Educ. Dev.* 35 1461–1482. 10.1080/10409289.2023.2292017

[B22] Cryer D. Tietze W. Burchinal M. Leal T. Palacios J. (1999). Predicting process quality from structural quality in preschool programs: a cross-country comparison. *Early Child. Res. Q.* 14 339–361. 10.1016/S0885-2006(99)00017-4

[B23] Curby T. W. Brock L. L. Hamre B. K. (2013). Teachers’ emotional support consistency predicts children’s achievement gains and social skills. *Early Educ. Dev.* 24 292–309. 10.1080/10409289.2012.665760

[B24] Dede Yildirim E. Frosch C. A. Santos A. J. Veríssimo M. Bub K. Vaughn B. E. (2024). Antecedents to and outcomes associated with teacher–child relationship perceptions in early childhood: further evidence for child-driven effects. *Child Dev.* 95 679–698. 10.1111/cdev.14033 37902065

[B25] Demaray M. K. Ruffalo S. L. Carlson J. Busse R. T. Olson A. E. McManus S. M. et al. (1995). Social skills assessment: a comparative evaluation of six published rating scales. *Sch. Psychol. Rev.* 24 648–671. 10.1080/02796015.1995.12085793

[B26] Denham S. A. Zahn-Waxler C. Cummings E. M. Iannotti R. J. (1991). Social competence in young children’s peer relations: patterns of development and change. *Child Psychiatry Hum. Dev.* 22 29–44. 10.1007/BF00706057 1748014

[B27] Diebold T. Perren S. (2022). Toddlers’ peer engagement in swiss childcare: contribution of individual and contextual characteristics. *Eur. J. Psychol. Educ.* 37 627–648. 10.1007/s10212-021-00552-2

[B28] Downer J. T. Booren L. M. Lima O. K. Luckner A. E. Pianta R. C. (2010). The individualized classroom assessment scoring system (inCLASS): preliminary reliability and validity of a system for observing preschoolers’ competence in classroom interactions. *Early Child. Res. Q.* 25 1–16. 10.1016/j.ecresq.2009.08.004 23175598 PMC3501735

[B29] Downes M. J. Brennan M. L. Williams H. C. Dean R. S. (2016). Development of a critical appraisal tool to assess the quality of cross-sectional studies (AXIS). *BMJ Open* 6:e011458. 10.1136/bmjopen-2016-011458 27932337 PMC5168618

[B30] Elizarov E. Czik A. Ziv Y. (2024). Kindergarten children’s academic engagement: a dual-pathway model including social information processing, social behavior in class, and teacher–child relationship quality. *Eur. J. Psychol. Educ.* 39 3729–3749. 10.1007/s10212-024-00803-y

[B31] Eslami A. A. Mazaheri M. A. Mostafavi F. (2014). Farsi version of social skills rating system-secondary student form: cultural adaptation, reliability and construct validity. *Iran J. Psychiatry Behav. Sci.* 8 97–104. 25053964 PMC4105611

[B32] Green K. D. Forehand R. (1980). Assessment of children’s social skills: a review of methods. *J. Behav. Assess.* 2 143–159. 10.1007/BF01338931

[B33] Hamre B. Hatfield B. Pianta R. Jamil F. (2014). Evidence for general and domain-specific elements of teacher–child interactions: associations with preschool children’s development. *Child Dev.* 85 1257–1274. 10.1111/cdev.12184 24255933

[B34] Hamre B. K. Pianta R. C. (2001). Early teacher–child relationships and the trajectory of children’s school outcomes through eighth grade. *Child Dev.* 72 625–638. 10.1111/1467-8624.00301 11333089

[B35] Hamre B. K. Pianta R. C. (2005). Can instructional and emotional support in the first-grade classroom make a difference for children at risk of school failure? *Child Dev.* 76 949–967. 10.1111/j.1467-8624.2005.00889.x 16149994

[B36] Hamre B. K. Pianta R. C. Downer J. T. DeCoster J. Mashburn A. J. Jones S. M. et al. (2013). Teaching through interactions: testing a developmental framework of teacher effectiveness in over 4,000 classrooms. *Elem. Sch. J.* 113 461–487. 10.1086/669616 34497425 PMC8423353

[B37] Hatfield B. E. Hestenes L. L. Kintner-Duffy V. L. O’Brien M. (2013). Classroom emotional support predicts differences in preschool children’s cortisol and alpha-amylase levels. *Early Childhood Res. Q.* 28 347–356. 10.1016/j.ecresq.2012.08.001

[B38] Higgins J. P. T. Thompson S. G. Deeks J. J. Altman D. G. (2003). Measuring inconsistency in meta-analyses. *BMJ* 327 557–560. 10.1136/bmj.327.7414.557 12958120 PMC192859

[B39] Hightower A. D. Work W. C. Cowen E. L. Lotyczewski B. S. Spinell A. P. Guare J. C. et al. (1986). The teacher-child rating scale: a brief objective measure of elementary children’s school problem behaviors and competencies. *Sch. Psychol. Rev.* 15 393–409. 10.1080/02796015.1986.12085242

[B40] Hoffman J. A. (2016). Promoting Healthy Social-Emotional Development in Vulnerable Young Children: The Importance of Head Start Teachers and Centers. Columbus, OH: The Ohio State University, 1–176.

[B41] Howes C. Hamilton C. E. (1993). The changing experience of child care: changes in teachers and in teacher-child relationships and children’s social competence with peers. *Early Child. Res. Q.* 8 15–32. 10.1016/S0885-2006(05)80096-1

[B42] Howes C. Rubin K. H. Ross H. S. French D. C. (1988). Peer interaction of young children. *Monogr. Soc. Res. Child Dev.* 53 1–92. 10.2307/11660623226426

[B43] Hunter J. E. Schmidt F. L. (2004). *Methods of Meta-Analysis: Correcting Error and Bias in Research Findings*, 2nd Edn. Thousand Oaks, CA: Sage. 10.1177/1094428106295494

[B44] Jaggy A.-K. Kalkusch I. Bossi C. B. Weiss B. Sticca F. Perren S. (2023). The impact of social pretend play on preschoolers’ social development: results of an experimental study. *Early Child. Res. Q.* 64 13–25. 10.1016/j.ecresq.2023.01.012

[B45] Józsa K. Oo T. Z. Borbélyová D. Podráczky J. (2025). Social skills development in children aged 4–8 years. *PLoS One* 20:e0332571. 10.1371/journal.pone.0332571 41004537 PMC12469092

[B46] Karam M. (2020). *The Influence of Mother-Child and Teacher-Child Interactions on Social Competence Development in Early Childhood.* Washington, DC: The Catholic University of America.

[B47] Kodak R. N. Güzel H. Ş (2024). Aggression among preschool children within the framework of temperament, attachment and parental attitudes. *Psikiyatr. Güncel Yaklaşımlar* 16 48–57. 10.18863/pgy.1213590

[B48] Landis J. R. Koch G. G. (1977). The measurement of observer agreement for categorical data. *Biometrics* 33 159–175. 10.2307/2529310 843571

[B49] Li L. Wang N. Zhang L. B. Zhang Y. Y. (2024). A three-level meta-analysis of the relationship between teacher-child interaction quality and preschool children’s academic abilities. *Stud. Presch. Educ.* 354 58–72. 10.13861/j.cnki.sece.2024.06.010

[B50] Li Y. F. (2020). The relationship between the quality of teacher-child interaction in kindergartens and young children’s social skills. *J. China Women’s Univ.* 32 74–81. 10.13277/j.cnki.jcwu.2020.01.013

[B51] Liu Q. Huang J. Caldwell M. P. Cheung S. K. Cheung H. Siu T. S. C. (2024). Gendered pathways to socioemotional competencies in very young children. *Sci. Rep.* 14:6360. 10.1038/s41598-024-56854-0 38493206 PMC10944455

[B52] Mashburn A. J. Pianta R. C. Hamre B. K. Downer J. T. Barbarin O. A. Bryant D. et al. (2008). Measures of classroom quality in prekindergarten and children’s development of academic, language, and social skills. *Child Dev.* 79 732–749. 10.1111/j.1467-8624.2008.01154.x 18489424

[B53] McClure E. B. (2000). A meta-analytic review of sex differences in facial expression processing and their development in infants, children, and adolescents. *Psychol. Bull.* 126 424–453. 10.1037/0033-2909.126.3.424 10825784

[B54] Michelle Taylor B. (2013). *Teacher-Child Interactions and Children’s Peer Engagement in Pre-Kindergarten.* Los Angeles, CA: University of California. DissERTATION

[B55] Mohamed A. H. H. (2018). Gender as a moderator of the association between teacher–child relationship and social skills in preschool. *Early Child Dev. Care* 188 1711–1725. 10.1080/03004430.2016.1278371

[B56] Muller K. (1989). Statistical power analysis for the behavioral sciences. *Technometrics* 31 499–500. 10.1080/00401706.1989.10488618

[B57] Pakarinen E. Lerkkanen M.-K. Von Suchodoletz A. (2020). Teacher emotional support in relation to social competence in preschool classrooms. *Int. J. Res. Method Educ.* 43 444–460. 10.1080/1743727X.2020.1791815

[B58] Partee A. M. Alamos P. Williford A. P. Downer J. T. (2022). Preschool children’s observed interactions with teachers: implications for understanding teacher–child relationships. *Sch. Ment. Health* 14 967–983. 10.1007/s12310-022-09517-2 36726649 PMC9886234

[B59] Perren S. Alsaker F. D. (2009). Depressive symptoms from kindergarten to early school age: longitudinal associations with social skills deficits and peer victimization. *Child Adolesc. Psychiatry Ment. Health* 3 28–38. 10.1186/1753-2000-3-28 19772574 PMC2754981

[B60] Pianta R. C. Hamre B. K. (2009). Conceptualization, measurement, and improvement of classroom processes: standardized observation can leverage capacity. *Educ. Res.* 38 109–119. 10.3102/0013189X09332374

[B61] Pianta R. C. Hofkens T. (2023). Defining early education quality using CLASS-observed teacher-student interaction. *Front. Psychol.* 14:1110419. 10.3389/fpsyg.2023.1110419 37519392 PMC10376698

[B62] Pianta R. C. Stuhlman M. W. (2004). Teacher-child relationships and children’s success in the first years of school. *Sch. Psychol. Rev.* 33 444–458. 10.1080/02796015.2004.12086261

[B63] Pires A. C. Rocha F. De Barros Neto A. J. Simão H. Nicolau H. Guerreiro T. (2020). “Exploring accessible programming with educators and visually impaired children,” in *Proceedings of the Interaction Design and Children Conference*, (London: ACM), 148–160. 10.1145/3392063.3394437

[B64] Reeve J. (2009). Why teachers adopt a controlling motivating style toward students and how they can become more autonomy supportive. *Educ. Psychol.* 44 159–175. 10.1080/00461520903028990

[B65] Rimm-Kaufman S. E. Curby T. W. Grimm K. J. Nathanson L. Brock L. L. (2009). The contribution of children’s self-regulation and classroom quality to children’s adaptive behaviors in the kindergarten classroom. *Dev. Psychol.* 45 958–972. 10.1037/a0015861 19586173

[B66] Roorda D. L. Koomen H. M. Y. Spilt J. L. Oort F. J. (2011). The influence of affective teacher-student relationships on students’ school engagement and achievement: a meta-analytic approach. *Rev. Educ. Res.* 81 493–529. 10.3102/0034654311421793

[B67] Salavera C. Usán P. Jarie L. (2020). Styles of humor and social skills in students, Gender differences. *Curr. Psychol.* 39 571–580. 10.1007/s12144-017-9770-x

[B68] Serena L. (2025). *An Ecological Approach to Emotion Socialization in the Early Childhood Classroom: Examining Contextual Influences on Children’s Early Social and Emotional Development.* Cambridge, MA: Harvard University.

[B69] Sette S. Spinrad T. L. Baumgartner E. (2013). Links among italian preschoolers’ socioemotional competence, teacher–child relationship quality, and peer acceptance. *Early Educ. Dev.* 24 851–864. 10.1080/10409289.2013.744684 24039375 PMC3769089

[B70] Siekkinen M. Pakarinen E. Lerkkanen M.-K. Poikkeus A.-M. Salminen J. Poskiparta E. et al. (2013). Social competence among 6-year-old children and classroom instructional support and teacher stress. *Early Educ. Dev.* 24 877–897. 10.1080/10409289.2013.745183

[B71] Slot P. L. Bleses D. (2018). Individual children’s interactions with teachers, peers, and tasks: the applicability of the inCLASS pre-K in danish preschools. *Learn. Individ. Differ.* 61 68–76. 10.1016/j.lindif.2017.11.003

[B72] Smith J. McLaughlin T. Aspden K. (2019). Teachers’ perspectives of children’s social behaviours in preschool: does gender matter? *Australas. J. Early Child.* 44 408–422. 10.1177/1836939119870889

[B73] Soininen V. Pakarinen E. Lerkkanen M.-K. (2023). Reciprocal associations among teacher–child interactions, teachers’ work engagement, and children’s social competence. *J. Appl. Dev. Psychol.* 85:101508. 10.1016/j.appdev.2022.101508

[B74] Son S.-H. C. Chang Y. E. (2018). Childcare experiences and early school outcomes: the mediating role of executive functions and emotionality. *Infant Child Dev.* 27:e2087. 10.1002/icd.2087

[B75] Song H. Zmyslinski-Seelig A. Kim J. Drent A. Victor A. Omori K. et al. (2014). Does facebook make you lonely?: a meta analysis. *Comput. Hum. Behav.* 36 446–452. 10.1016/j.chb.2014.04.011

[B76] Spilt J. L. Koomen H. M. Y. (2022). Three decades of research on individual teacher-child relationships: a chronological review of prominent attachment-based themes. *Front. Educ.* 7:920985. 10.3389/feduc.2022.920985

[B77] Sroufe L. A. (2005). Attachment and development: a prospective, longitudinal study from birth to adulthood. *Attach. Hum. Dev.* 7 349–367. 10.1080/14616730500365928 16332580

[B78] Stephens C. M. Crosby D. A. Yaya-Bryson D. Reid A. (2023). Supporting spanish-english DLLs in head start: peer language match, instructional language match, and emotional support as predictors of approaches to learning and social skills. *Early Child. Res. Q.* 63 121–132. 10.1016/j.ecresq.2022.11.005

[B79] Stillerova L. (2018). *Bidirectional associations between Classroom Climate and Children’s Social–Emotional Skills and Problem Behaviors in Preschool.* Fairfax, VA: George Mason University.

[B80] Tanya M. Paes R. J. Duncan D. J. Purpura Schmitt S. A. (2021). “The association between preschool teacher-child relationship and children’s kindergarten outcomes,” in *Poster Presented at the CogSci 2021 Conference*, (London).

[B81] Tobin J. (2005). Quality in early childhood education: an anthropologist’s perspective. *Early Educ. Dev.* 16 421–434. 10.1207/s15566935eed1604_3 42317603

[B82] Tutkun C. Eskidemir Meral S. (2025). Preschool children’s social skills, problem behaviors, academic self-esteem and teacher-child relationship: a serial mediation model. *Front. Psychol.* 16:1453193. 10.3389/fpsyg.2025.1453193 40166393 PMC11955605

[B83] Verschueren K. Koomen H. M. Y. (2012). Teacher–child relationships from an attachment perspective. *Attach. Hum. Dev.* 14 205–211. 10.1080/14616734.2012.672260 22537520

[B84] Vitiello V. E. Nguyen T. Ruzek E. Pianta R. C. Whittaker J. V. (2022). Differences between Pre-K and Kindergarten classroom experiences: Do they predict children’s social-emotional skills and self-regulation across the transition to kindergarten? *Early Child. Res. Q.* 59 287–299. 10.1016/j.ecresq.2021.11.009

[B85] Vygotskiĭ L. S. Cole M. (1978). *Mind in Society: the Development of Higher Psychological Processes.* Cambridge, MA: Harvard University Press.

[B86] Wang S. Hu B. Y. LoCasale-Crouch J. Li J. (2021). Supportive parenting and social and behavioral development: Does classroom emotional support moderate? *J. Appl. Dev. Psychol.* 77:101331. 10.1016/j.appdev.2021.101331

[B87] Wang Y. Tao Y. Zhu L. Li Y. Huang D. (2022). Preschool children’s negative affect and social skills in China: the moderating effect of the teacher–child relationship. *Front. Psychol.* 13:991039. 10.3389/fpsyg.2022.991039 36211844 PMC9533076

[B88] Whittaker J. E. Hofkens T. Vitiello V. E. Pianta R. C. DeCoster J. Ansari A. (2024). Patterns of children’s readiness at school entry and their association with kindergarten academic and social-emotional outcomes: Do classroom interactions matter? *Early Child. Res. Q.* 66 112–123. 10.1016/j.ecresq.2023.09.005

[B89] Williford A. P. Maier M. F. Downer J. T. Pianta R. C. Howes C. (2013a). Understanding how children’s engagement and teachers’ interactions combine to predict school readiness. *J. Appl. Dev. Psychol.* 34 299–309. 10.1016/j.appdev.2013.05.002 26722137 PMC4694586

[B90] Williford A. P. Vick Whittaker J. E. Vitiello V. E. Downer J. T. (2013b). Children’s engagement within the preschool classroom and their development of self-regulation. *Early Educ. Dev.* 24 162–187. 10.1080/10409289.2011.628270 23441104 PMC3579638

[B91] Wu P. P. (2023). *The Relationship between Teachers’ Social-Emotional Competence and Social Development of children aged 3–6: The Mediating Role of Teacher-Child Interaction (Master’s thesis).* Guilin: Guangxi Normal University.

[B92] Wu Y. Fang M. Wu J. Chen Y. Li H. (2022). Shyness and school engagement in Chinese suburban preschoolers: a moderated mediation model of teacher–child closeness and child gender. *Int. J. Environ. Res. Public Health* 19:4270. 10.3390/ijerph19074270 35409950 PMC8998169

[B93] Xu C. Huizinga M. De Luca G. Pollé S. Liang R. Sankalaite S. et al. (2023). Cultural universality and specificity of teacher-student relationship: a qualitative study in belgian, Chinese, and italian primary school teachers. *Front. Psychol.* 14:1287511. 10.3389/fpsyg.2023.1287511 38034285 PMC10682107

[B94] Xu C. Huizinga M. Tekelia Ekubagewargies D. Soetaert J. Van Den Noortgate W. Baeyens D. (2024). The relation between teacher–student interaction and executive function performance in children: a cross-cultural meta-analysis. *Educ. Psychol.* 59 195–215. 10.1080/00461520.2024.2315527

[B95] Yu X. W. Zhao Y. M. Jin F. (2022). The influence of teacher-child interaction quality on self-control ability of 3- to 6-year-old children. *Early Childhood Educ.* 64–68 91.

[B96] Zhao H. (2018). *Examining Contributors to Preschoolers’ Classroom Engagement using Structural Equation Modeling.* Johnson: East Tennessee State University, 1–198.

[B97] Zhao Y. M. (2020). *The Relationship between Teacher-Child Interaction Quality and Young Children’s Self-Control.* Liaoning: Shenyang Normal University.

[B98] Zhu J. Xia X. Wu Q. Zou S. Li Y. (2023). Callous-unemotional traits and social adjustment among chinese preschoolers: the moderating role of teacher-child relationship. *Int. J. Environ. Res. Public Health* 20:3426. 10.3390/ijerph20043426 36834123 PMC9966528

[B99] Zhu Z. Jiao D. Li X. Zhu Y. Kim C. Ajmal A. et al. (2023). Measurement invariance and country difference in children’s social skills development: evidence from Japanese and Chinese samples. *Curr. Psychol.* 42 20385–20396. 10.1007/s12144-022-03171-2 35531072 PMC9061028

[B100] Zhuang Y. Y. (2019). *The Influence of Teacher-Child Interaction Quality on Social Development of Young Children from Different Family Socioeconomic Statuses.* Changchun: Northeast Normal University.

