# Supplementary material for: A meta-analysis of the association between teacher-child interaction quality and young children’s social skills
Source: Front Psychol. 2026 Jul 9;17:1808031. doi: 10.3389/fpsyg.2026.1808031 (PMC13391879; doi:10.3389/fpsyg.2026.1808031)
Supplement: Supplementary file 8 [file Table_8.docx]

Coding Manual

This manual describes the variable definitions, coding rules, and procedures used for data extraction and effect size aggregation.

1. Variable Definitions

(1) Teacher–Child Interaction Quality (Predictor)

Three subdimensions: Emotional Support; Instructional Support; Classroom Organization

(2) Children’s Social Skills (Outcome)

Included subdomains: communication; cooperation; assertion; responsibility; empathy; engagement; participation; self-control

2. Moderator Variables and Coding Rules

(1) Child mean age: Continuous value (years) reported in each study.

(2) Percentage of male children: Continuous value (%) reported in each study.

(3) Cultural region: Europe、Middle East、East Asia

(4) Informant type for Teacher–Child Interaction: Observer-rated; Teacher-reported

(5) Measurement tool for Teacher–Child Interaction: The measurement tools for teacher-child interaction quality identified in the included studies are as follows:

CLASS, 2008 / CLASS-ECER

Classroom Assessment Scoring System, a widely used observational instrument for assessing the quality of teacher-child interactions in early childhood classrooms, covering emotional support, classroom organization, and instructional support domains.

inCLASS, 2010 / inCLASS, 2012

Individualized Classroom Assessment Scoring System, an observational tool focusing on child-level interactions with teachers and peers.

STRS, 2001 / STRS, 1992

Student-Teacher Relationship Scale, a teacher-reported measure assessing the quality of teacher-child relationships, including closeness, conflict, and dependency.

M-ORCE

Mother–Child Observation Rating Scale for Early Childhood, an observational tool designed to assess the quality of mother-child interactions in early childhood settings.

TCR

Teacher-Child Relationship scale, a measure of the affective quality of the teacher-child relationship.

(6) Measurement tool for Social Skills

The measurement tools for children’s social skills identified in the included studies are as follows:

PBRS (Positive Behavior Rating Scale)

A scale designed to assess children’s positive social behaviors, including cooperation, prosocial actions, and appropriate interaction skills.

TCRS (Teacher–Child Relationship Scale)

A teacher-reported measure originally designed to assess teacher-child relationship quality, which is also used to capture social-emotional aspects of children’s behavior.

SSRS (Social Skills Rating System)

A widely used multi-informant scale to rate children’s social skills (e.g., cooperation, assertion, responsibility) and problem behaviors in school and home settings.

SSIS (Social Skills Improvement System)

A revised, comprehensive rating system that assesses both social skills and problem behaviors, including cooperation, empathy, self-control, and communication.

BITSEA (Brief Infant Toddler Social and Emotional Assessment)

A screening tool for assessing social-emotional development and behavior problems in infants and toddlers, including social competence and regulation skills.

MASCS (Multisource Assessment of Social Competence Scale)

A multi-source scale designed to evaluate children’s social competence using information from multiple raters (e.g., teachers, peers, observers).

ASQ (Ages and Stages Questionnaires)

A developmental screening tool that includes items assessing social-emotional development across different age ranges.

SOCOMP (Self- and Other-oriented social Competences)

A scale assessing children’s social competence, covering both self-oriented (e.g., self-control) and other-oriented (e.g., cooperation, empathy) social skills.

SCBE (Social Competence and Behavior Evaluation)

A scale designed to assess social competence, emotional regulation, and problem behaviors in young children.

CBS (Child Behavior Scale)

A scale measuring a range of children’s behaviors, including social skills, conduct problems, and emotional adjustment.

SDQ (Strengths and Difficulties Questionnaire)

A brief behavioral screening questionnaire assessing prosocial behavior and emotional/behavioural difficulties, widely used to measure social skills and adjustment.

3. Missing Data Handling: Missing values were not imputed. Only data explicitly reported in original studies were used. Studies with missing moderator values were excluded from corresponding moderator analyses.

4. Effect Size Coding & Aggregation:

Correlation coefficients (r) were used as the main effect size;

β coefficients were converted to r using the formula: r = β × 0.98 + 0.05 (β ≥ 0); r = β × 0.98 – 0.05 (β < 0)

Multiple dependent effect sizes within one study were aggregated using arithmetic mean or Hunter–Schmidt composite method.

One independent effect size per study was retained.
