# Supplementary material for: A meta-analysis of the association between teacher-child interaction quality and young children’s social skills
Source: Front Psychol. 2026 Jul 9;17:1808031. doi: 10.3389/fpsyg.2026.1808031 (PMC13391879; doi:10.3389/fpsyg.2026.1808031)
Supplement: Supplementary file 9 [file Data_Sheet_1.pdf]

S 1 Supplementary Table

Author and Year	OES 1	OES 2	OES 3	Aggregation Rule	Final Included Effect Size
Lucia,2018	T1 = 0.17	T2 = 0.16	——	Arithmetic mean	r=0.17
Andrew,2008	r=0.1088	r=0.0696	——	Arithmetic mean	r=0.089
Shuang,2021	r=0.32	——	——	Single effect size	r=0.320
Baldanza,2013	r=0.083	r=0.004	r=0.186	Arithmetic mean	r=0.091
Ahmed,2017	r=0.53	r=0.31	r=0.22	Hunter aggregation	r=0.440
Martine,2015	T1 r=-0.04	——	——	Single effect size	r=-0.04
Ana,2016	r=0.07	r=-0.02	r=0.08	Hunter aggregation	r=0.070
Timothy,2012	R=0.1	——	——	Single effect size	r=0.100
Margaret,2008	ECERS r=0.09	CLASS：r=0.02	——	Arithmetic mean	r=0.060
Jill,2016	r=0.100	——	——	Single effect size	r=0.100
Virginia,2021	T1 r=0.3342	T2 r=0.2205	——	Arithmetic mean	r=0.280
Meredith,2020	r=0.510	——	——	Single effect size	r=0.510
Pauline L，2018	r=-0.17	——	——	Single effect size	r=-0.170
Elif,2024	r=0.126	——	——	Single effect size	r=0.126
Christina,2022	r=-0.01	——	——	Single effect size	r=- 0.010
Greensboro,2009	r=0.138	——	——	Single effect size	r=0.138
Tatiana,2021	r=0.031	r=0.053	——	Arithmetic mean	r=0.042
Nadine,2023	T1 r=-0.1	T3 r=-0.075	——	Arithmetic mean	r=-0.090
Margaret,2020	r=0.11	——	——	Single effect size	r=0.110
Eija,2020	T1 r=0.17	T2 r=0.465	——	Arithmetic mean	r=0.320
Krystal,2019	r=-0.050	——	——	Single effect size	r=-0.050
Jessica,2023	r=0.02	——	——	Single effect size	r=0.020
Martti,2013	r=-0.125	r=0.167	——	Arithmetic mean	r=0.021
Laura,2014	r=0.51	r=0.2	——	Arithmetic mean	r=0.360
Holly,2007	r=0.51	——	——	Single effect size	r=0.510
Li,2020	r=0.287	r=0.261	r=0.189	Arithmetic mean	r=0.246
Zhang,2019	r=0.023	——	——	Single effect size	r=0.023
Yu,2022	r = 0.098<br>r = 0.105<br>r = 0.175	r = 0.359<br>r = 0.489<br>r = 0.579<br>r = 0.401	r = 0.402<br>r = 0.57<br>r = 0.562	Hunter aggregation	r=0.518
Wu,2020	r=0.439	r=0.29	r=0.395	Hunter aggregation	r=0.459
Sara,2009	r=0.12<br>r=0.25<br>r=0.1	r=-0.02<br>r=0.15<br>r=-0.03	——	Hunter aggregation	r=0.150
Amanda,2013	r=0.27	——	——	Single effect size	r=0.270
Zhu,2023	r=0.55	——	——	Single effect size	r=0.550
Zhao,2018	r=0.576	r=0.490	——	Arithmetic mean	r=0.533
Einat,2024	R=0.32	——	——	Single effect size	r=0.320
Wang,2022	r=0.38	r=0.29	r=0.40	Hunter aggregation	r=0.380
Budrevich,2019	Fall r=0.026	Spring r=0.03	——	Hunter aggregation	r=0.038
CogSci,2021	r=0.26	r=0.5	——	Arithmetic mean	r=0.380

Stefania,2013	r=0.40	——	——	Single effect size	r=0.400
Lily,2025	r=0.18	——	——	Single effect size	r=0.180
Liu,2024	r=0.26	——	——	Single effect size	r=0.260
Cansu,2025	r=0.47	——	——	Single effect size	r=0.470
Wu，2022	r=0.359	r=0.187	——	Arithmetic mean	r=0.273

Note. Author and Year = First author and publication date; OES1/OES2/OES3 = Original Effect Size 1/2/3, referring to raw correlation coefficients rep orted in the primary study. Labels (e.g., T1, T2, ECERS, CLASS) indicate different measurement occasions, subdimensions, or tools; Aggregation rule s: Single effect size: The only reported effect size was used directly; Arithmetic mean: Multiple effect sizes from the same study measuring the same c onstruct were averaged, As an example, for Lucia (2018), which reported two effect sizes r_{T1} = 0.17, r_{T2} = 0.16), the arithmetic mean was calculated as follows: Step 1: 0.17 + 0.16 = 0.33; Step 2: 0.33 / 2 = 0.165; Step 3: rounded to two decimal places, r = 0.17; Hunter-Schmidt (2004) aggregation: Multiple dependent effect sizes across different subdimensions were combined using the Hunter-Schmidt reliability correction formula, Example of Hu nter-Schmidt aggregation (Yu, 2022):

Step 1: Sum the correlation coefficients across 10 dimensions:

0.098 + 0.105 + 0.175 + 0.359 + 0.489 + 0.579 + 0.401 + 0.402 + 0.570 + 0.562 = 3.74

Step 2: Calculate the mean intercorrelation of the 10 dimensions ($\overline{r_{xx}}$):

Based on the total pairwise correlation sum of 21.088 across 45 pairs, $\overline{r_{xx}}$ = 21.088 ÷ 45 ≈ 0.4686

Step 3: Compute the denominator of the Hunter-Schmidt formula:$\sqrt{10 + 10 \times 9 \times 0.4686}$ = $\sqrt{52.176}$ ≈ 7.2233

Step 4: Derive the final composite effect size:r_{xy} = 3.74 ÷ 7.2233 ≈ 0.518.

Final Included Effect Size = The aggregated independent effect size used in the meta-analysis.

Hunter-Schmidt (2004) aggregation: $r_{xy_b} = \frac{\Sigma r_i r_j}{\sqrt{n+n(n-1)\overline{r_{x_i x_j}}}\sqrt{m+m(m-1)\overline{r_{y_i y_j}}}}$; r_{xy}= aggregated combined effect size between the focal constructs; r_{xiyi}= correlation coefficient between the i-th measure of the predictor and the j-th measure of the outcome; m = number of dependent measures of the predictor; n = number of dependent measures of the outcome; $\bar{r}_{xx}$= mean correlation between subdimensions of the predictor; $\bar{r}_{yy}$= mean correlation between sub dimensions of the outcome. Full details of effect size coding and aggregation procedures for each included study are provided in Supplementary Table S2.
